# Effect of insulin infusion line on glycaemic variability in a perioperative high dependency unit (HDU): a prospective randomised controlled trial

**DOI:** 10.1186/s13613-017-0298-x

**Published:** 2017-07-11

**Authors:** Stéphanie Genay, Bertrand Décaudin, Sabine Ethgen, Arnaud Alluin, Elodie Babol, Julien Labreuche, Hélène Behal, Marie-Christine Vantyghem, Pascal Odou, Gilles Lebuffe

**Affiliations:** 1 EA 7365 - GRITA - Groupe de Recherche sur les formes Injectables et les Technologies Associées, University of Lille, CHU Lille, 59000 Lille, France; 2 Institut de Pharmacie, CHU Lille, 59000 Lille, France; 3 Département d’anesthésie-réanimation, CHU Lille, 59000 Lille, France; 4 EA 2694 - Santé publique: épidémiologie et qualité des soins, University of Lille, CHU Lille, 59000 Lille, France; 5 Service d’Endocrinologie et Métabolisme, INSERM U1190, European Genomics Institute for Diabetes EGID, CHU Lille, 59000 Lille, France; 6 Faculté de Pharmacie, 3, Rue du Professeur Laguesse, BP 83, 59006 Lille Cedex, France

**Keywords:** Insulin, Perioperative care, Infusions, intravenous/instrumentation, Infusion pumps, Drug delivery systems/instrumentation

## Abstract

**Background:**

Glucose control is an important issue in post-operative patients. The objective here was to compare two insulin infusion lines by syringe pumps to assess the impact of medical devices on glycaemic variability in surgical patients under intensive insulin therapy. This open, prospective, single-centre randomised study was conducted in a fifteen-bed perioperative high dependency unit (HDU) in a university hospital. In total, 172 eligible patients receiving insulin therapy agreed to participate in the study. Subcutaneous continuous glucose monitoring was set up for all patients and an optimised system with a dedicated insulin infusion line for half of the patients.

**Results:**

Eighty-six patients were infused via the optimised infusion line and 86 patients via the standard infusion line. No significant difference was found according to the glycaemic lability index score [mean difference between groups (95% CI): −0.09 (−0.34; 0.16), *p* = 0.49 after multiple imputation]. A glucose control monitoring system indicated a trend towards differences in the duration of hypoglycaemia (blood glucose level below 70 mg dl^−1^ (3.9 mmol l^−1^) over 1000 h of insulin infusion (9.7 ± 25.0 h in the standard group versus 4.4 ± 14.8 h in the optimised group, *p* = 0.059) and in the number of patients experiencing at least one hypoglycaemia incident (25.7 vs. 12.9%, *p* = 0.052). Time in the target range was similar for both groups.

**Conclusions:**

The use of optimised infusion line with a dedicated insulin infusion line did not reduce glycaemic variability but minimised the incidence of hypoglycaemia events. The choice of the medical devices used to infuse insulin seems important for improving the safety of insulin infusion in perioperative HDU.

## Background

Glucose control is an important issue in post-operative patients. In 2001, special attention was paid to glycaemic variability. Although tight glycaemic control improved the outcome of critically ill patients in a surgical intensive care unit (ICU) [1], the NICE-SUGAR study demonstrated that intensive glucose control increased mortality when compared to conventional glucose control [2]. Patients require continuous intravenous insulin infusion to control glucose level, but intensive insulin therapy (IIT) is often associated with an increased risk of hypoglycaemia [3, 4]. A recent study contrasted the importance of glycaemic control in critically ill patients with the occurrence of hypoglycaemia which impacts on the mortality of diabetics in general, but especially of those at risk [5, 6].

In the above works, the issue of insulin administration methods was not addressed. Yet, many parameters can affect the drug delivery of narrow therapeutics where multiple infusions are involved, as in ICUs. The main parameter to be considered seems to be the common space volume within the infusion line between drugs simultaneously infused, as shown by Lovich et al. [7, 8]. The time to reach a steady state after initiating or ceasing drug infusion is considerably reduced with a low common volume manifold compared to a high volume design [9]. Several studies have shown the benefits of a reduction in infusion line common volume in predicting drug delivery and so avoiding incidents such as unexpected drug bolus [7–16].

In the case of insulin, rationalisation of the infusion system could eliminate the unwanted insulin bolus that causes hypoglycaemic events. This approach was studied by Maury et al. [17] in a retrospective study in a medical ICU, and thanks to an optimised insulin infusion line, they were able to limit hypoglycaemia occurrence during IIT. This work opened the way to our prospective study assessing the impact of such a system on glycaemic control and variability.

Two tubing systems of insulin infusion by syringe pumps were compared to assess the impact of medical devices on glycaemic variability in surgical patients under IIT admitted to a high dependency unit (HDU). The evolution in patients’ blood glucose levels was followed in two ways: by glucometer and by continuous glucose monitoring (CGM), the latter being more accurate as it is continuous [18].

Our main objective was to determine whether an optimised infusion line for insulin therapy had any impact on glycaemic variability and patient outcome. Secondary objectives were to study the effects of other blood glucose parameters.

## Methods

### Protocol

This open, prospective, single-centre randomised study was performed in a perioperative HDU at the Lille University Hospital between September 2012 and December 2013. The perioperative HDU has 15 beds and admits all surgical patients requiring organ support after vascular, endocrine and digestive surgery with an average annual admission rate of 1076 patients. The protocol was approved by the patients’ protection committee (CPP Nord-Ouest IV) and registered with the US clinical trials database (ClinicalTrials.gov ID NCT02812927) and with the French medicine agency (ID-RCB number 2012-A00188-35, ANSM). Signed informed consent was obtained from patients or next of kin before randomisation, in conformity with national regulations. The study sponsor had no role in supervising the study or interpreting data.

### Patients

Inclusion criteria were: age from 18 to 80; patients undergoing elective or emergency vascular or abdominal surgery and requiring post-operative admission to the HDU; treatment with insulin on a bi-lumen central venous catheter for more than 48 h; eligibility for interstitial glucose monitoring; and blood glucose control every 3 h.

Exclusion criteria were: pregnant or breastfeeding women; patients unwilling to participate in the study or participating in another biomedical study; patients unable to understand the study and its objectives or under guardianship; patients malnourished (BMI < 18 kg m^−2^) or with morbid obesity (BMI > 40 kg m^−2^); patients in shock (septic or haemodynamic); and patients refusing to sign the Medtronic consent on the storage of personal data.

### Measures

Blood glucose was measured by two methods: the first by drawing samples from the fingertip using a glucometer device (StatStrip Xpress, Nova Biomedical, Les Ulis, France) every 3 h and for the second another medical device (iPro2, Medtronic, Boulogne-Billancourt, France) was used to continuously and blindly record interstitial glucose level. Insulin therapy was adapted only from blood glucose levels read on the glucometer according to HDU protocol. CGM data were used blindly only after removing the patient’s CGM.

The primary endpoint was the glycaemic lability index (GLI), which assesses glycaemic variability [19].

The secondary endpoints were: mean blood glucose; glucose variability assessed by the standard deviation (SD) of blood glucose (BG) values and the mean amplitude glycaemic excursions (MAGE) score; hypoglycaemic incidence and events; time spent in the hypoglycaemic/hyperglycaemic target range; amount of insulin; number of interventions on the infusion line (i.e. start/stop of drug infusion and change in flow rate); and number of insulin flow rate changes.

Hypoglycaemia was defined as blood glucose level below 70 mg dl^−1^ (3.9 mmol l^−1^), hyperglycaemia as over 180 mg dl^−1^ (10.0 mmol l^−1^). The rate of hypoglycaemic or hyperglycaemic events was defined as the percentage of patients experiencing at least one episode of either detected by one of the two glucose control devices. The time spent in hypoglycaemia, hyperglycaemia or in the target range per 1000 h of insulin therapy was determined from iPro2 data. The mean and SD of BG were calculated from the data of the two glucose control devices. GLI and MAGE were calculated from the glucometer data.

Patient outcome was recorded until discharge from hospital or until the 28th day after HDU admission if the patient had not been discharged before this day.

### Study design

Upon HDU admission, patients were randomly assigned in a ratio of 1:1 to be infused by the conventional insulin infusion system or by the optimised system with dedicated insulin infusion. Randomisation was carried out using sequentially numbered, sealed, opaque envelopes containing treatment group assignments obtained from computer-generated random numbers provided by an independent statistician. The patient was declared randomised when the seal was broken. The HDU pharmacist opened the envelope when the patient left the operating room and was directly hospitalised in the HDU unit. After randomisation, the HDU nurse set up the appropriate infusion line and the iPro2 device.

The protocol was implemented for at least 48 h or until the end of insulin therapy.

Regular human insulin (1 IU ml^−1^) was administered by continuous intravenous infusion via syringe pumps. Infusion was systematically started after surgery and adjusted according to patient blood glucose level: 1 IU h^−1^ between 80 (4.4) and 120 (6.7) mg dl^−1^ (mmol L^−1^), 2 IU h^−1^ between 121 (6.7) and 180 (10.0) mg dl^−1^ (mmol l^−1^), 3 IU h^−1^ between 181 (10.0) and 220 (12.2) mg dl^−1^ (mmol l^−1^), 4 IU h^−1^ between 221 (12.2) and 300 (16.7) mg dL^−1^ (mmol l^−1^) and 5 IU h^−1^ ≥ 301 (16.7) mg dl^−1^ (mmol l^−1^). Insulin infusion was stopped when blood glucose was ≤80 (4.4) mg dl^−1^ (mmol l^−1^) and 30% dextrose was given when blood glucose was ≤60 (3.3) mg dl^−1^ (mmol l^−1^).

**Fig. 1:**
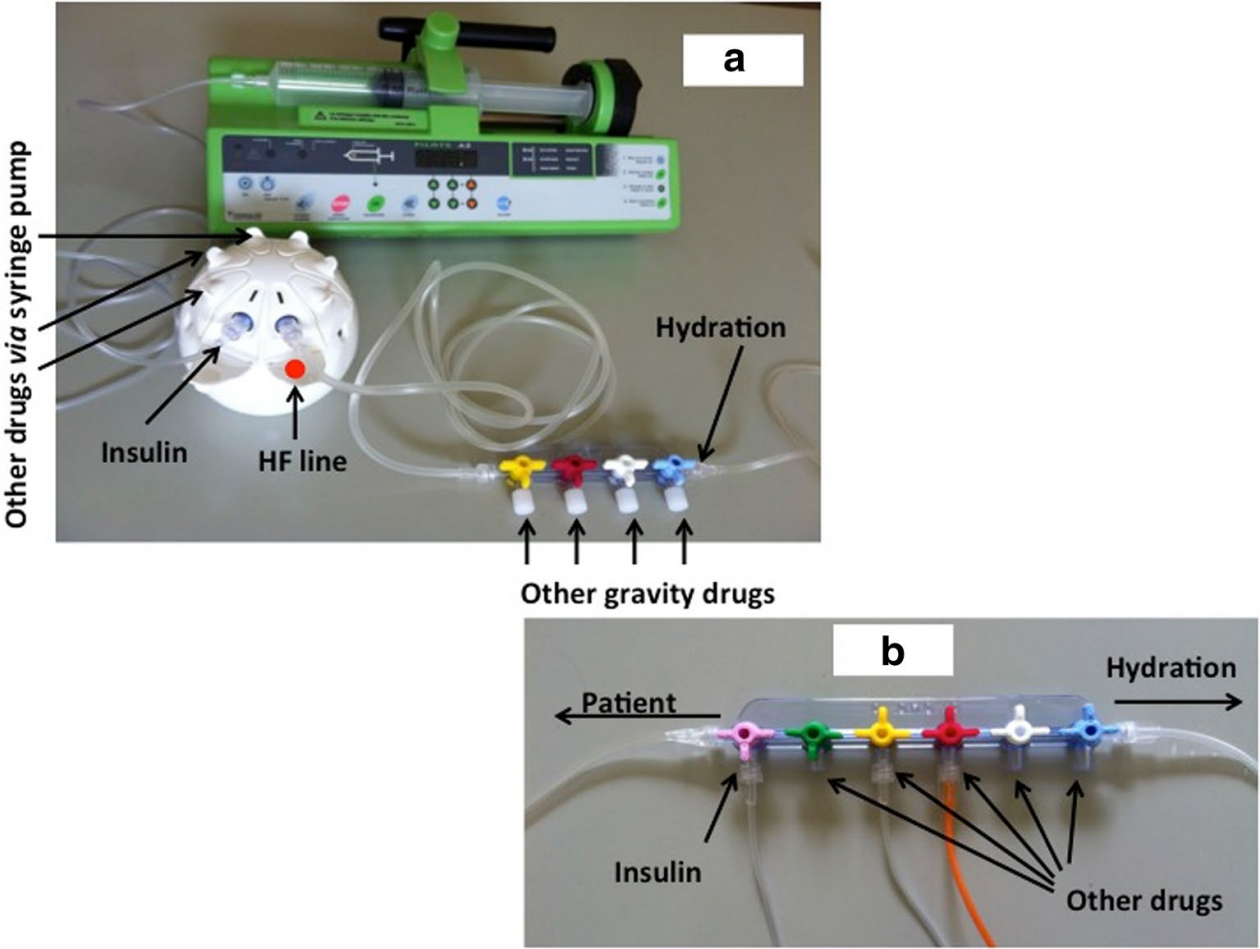
Schematic representation of the two infusion lines assessed. **a** Standard insulin infusion line using a six-stopcock manifold connected to the distal line of a multilumen central venous catheter by 150 cm tubing (RPB6315, Cair LGL, Lissieu, France). **b** Optimised insulin infusion line using a multilumen device (Edelvaiss Multiline-8, Doran International, Toussieu, France)

The standard insulin infusion line consisted of a six-stopcock manifold connected to the distal port of a multilumen central venous catheter by 150 cm tubing with an internal diameter of 2.5 mm (RPB6315, Cair LGL, Lissieu, France) (Fig. 1a). The common volume between infused drugs was roughly 8.5 ml. Insulin was systematically infused via the proximal port of the manifold; carrier was infused through the manifold via a pump; all other drugs were infused through the other five stopcocks.

The optimised insulin infusion line was a multilumen device (Edelvaiss Multiline-8, Doran International, Toussieu, France) (Fig. 1b) [14]. This device has ports for eight infusions, which run through separate channels within a 150 cm flexible plastic tube. Fluids from the individual channels do not meet until they exit the distal tip (dead volume of 0.9 ml). Carrier was infused through the high flow (HF) line (dead volume of 2.9 ml), and insulin was infused systematically next to the HF line port. All other drugs were administered via adjacent ports on the Multiline-8. The common volume between infused drugs was reduced to that of the internal lumen alone of the central venous catheter.

### Statistical analysis

The study was designed to have a statistical power of 80% with a two-sided alpha level of 0.05 to show the superiority of the optimised infusion line over the standard line in controlling glycaemic variability, assessed by GLI (primary endpoint). Sample size was calculated using the mean (±standard deviation, SD) of GLI assessed in 26 consecutive patients treated at our HDU centre with the standard insulin infusion system. In this unpublished cohort, the mean ± SD of GLI was 1070 ± 743 (mg dl^−1^)^2^/hj^−1^. Assuming an improvement of 30% in GLI values with the optimised infusion line (corresponding to a mean difference of 321), we estimated that 86 patients per group (a total of 172 patients) were necessary.

Analyses were performed on all patients in their original randomised group (respecting the intention-to-treat principle). Qualitative variables were expressed as frequencies and percentages. Quantitative variables were expressed as mean ± SD and medians with interquartile range. Normality of distribution was assessed graphically and using the Shapiro–Wilk test.

All analyses of GLI values (primary endpoint) were performed on log-transformed values in view of skewed distribution. Any difference between groups was calculated as a mean difference (optimised infusion line versus standard line) with a 95% confidence interval and tested using the Student *t* test. Missing data for the primary endpoint were treated by multiple imputations, using a regression-switching approach (chained equation with *m* = 20 imputations obtained with the R statistical software version 3.03). Imputation procedure was performed under the missing-at-random assumption, using all patients’ characteristics on inclusion as covariates (see Table 1) with a predictive mean matching method for continuous variables and logistic regression models (binary, ordinal or polynomial) for qualitative variables [20]. Estimates from each imputed dataset were combined using Rubin’s rules [21]. Complete case analysis was performed as a sensitivity analysis.

**Table 1:** Patients baseline characteristics according to study group

Parameters	Standard infusion line (*n* = 86)	Optimised infusion line (*n* = 86)
Age (years)	62.6 ± 10.3 (63.0)	61.8 ± 10.5 (61.0)
BMI (kg.m^−2^)	25.5 ± 5.2 (25.0)	25.5 ± 4.9 (25.9)
Men, *n* (%)	33 (38.4)	27 (31.4)
ASA score, *n* (%)		
ASA 1	11 (12.8)	17 (19.8)
ASA 2	56 (65.1)	55 (63.9)
ASA 3	18 (20.9)	13 (15.1)
ASA 4	1 (1.2)	1 (1.2)
Number of CRFs, *n* (%)		
0	14 (17.5)	9 (11.0)
1	10 (25.0)	23 (28.0)
2	9 (11.3)	20 (24.4)
3	37 (46.2)	30 (36.6)
Dyslipidemia, *n* (%)	30 (37.5)	27 (32.9)
High blood pressure, *n* (%)	37 (46.3)	39 (47.6)
Pulmonary history, *n* (%)	11 (13.8)	15 (18.3)
Cirrhosis history, *n* (%)	8 (10.0)	4 (4.9)
Liver surgery history, *n* (%)	8 (10.0)	6 (7.3)
Pancreas disease history, *n* (%)	4 (5.0)	3 (3.7)
Corticosteroid therapy, *n* (%)	1 (1.3)	4 (4.9)
Kidney disease history, *n* (%)	2 (2.5)	3 (3.7)
Blood disease history, *n* (%)	2 (2.5)	2 (2.4)
Pre-existing diabetes, *n* (%)	17 (21.3)	12 (14.6)
Scheduled surgery, *n* (%)	85 (98.8)	84 (97.7)
Type of surgery, *n* (%)		
Esophagus	33 (41.3)	34 (41.5)
Liver	29 (36.3)	31 (37.8)
Pancreas	8 (10.0)	8 (9.8)
Vascular and general	10 (12.5)	9 (11.0)
SAPS II	22.2 ± 8.4 (23.0)	21.8 ± 7.9 (21.0)
SOFA score	1.3 ± 1.8 (0.0)	1.2 ± 1.7 (0.0)

Values are expressed as mean ± SD (median) unless otherwise indicated

*ASA* Physical status score defined by the American Society of Anesthesiologists, *BMI* body mass index, *CRFs* cardiovascular risk factors, *SAPS II* simplified acute physiological score, *SD* standard deviation, *SOFA* sequential organ failure assessment

Between-group comparisons for secondary endpoints were made using the Chi-square test (or Fisher’s exact test when the expected cell frequency was <5) for qualitative endpoints and the Student *t* test (or Mann–Whitney U test in the case of non-Gaussian distribution) for quantitative endpoints. No adjustment was made for multiple comparisons.

Statistical testing was performed at the two-tailed *α* level of 0.05. Data were analysed using the SAS software package, release 9.3 (SAS Institute, Cary, NC).

## Results

**Fig. 2:**
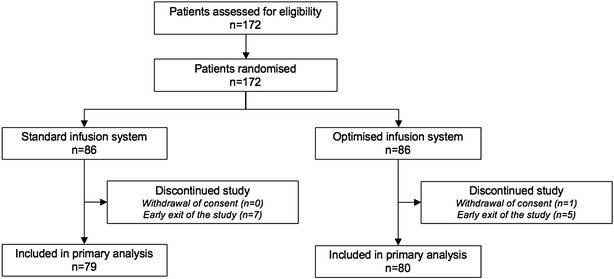
Study flow chart

A total of 172 eligible patients accepted the protocol and agreed to participate in the study: 86 patients were treated with the optimised infusion line and 86 patients the standard infusion line (Fig. 2).

### Baseline characteristics

Baseline characteristics were well balanced over the two study groups (Table 1). Before entering the HDU, patients had undergone mainly oesophageal and hepatic surgery; 78% in a context of scheduled surgery.

### Glycaemic outcomes

**Fig. 3:**
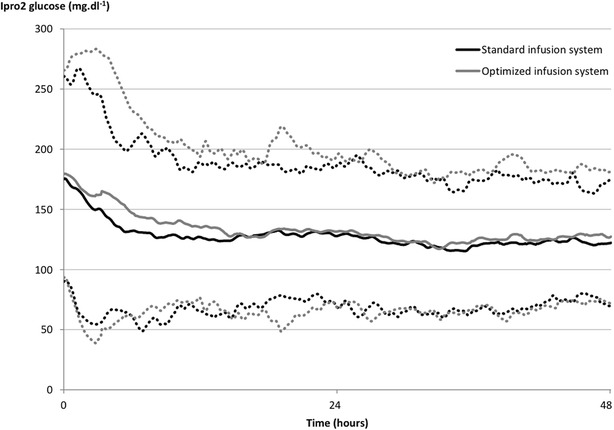
Mean ±2 standard deviations of blood glucose values measured by iPro2 over 48 h according to the insulin infusion lines

No significant difference was found between the two groups as regards GLI score (mean difference between groups (95% CI): −0.09 (−0.34; 0.16), *p* = 0.49 after multiple imputation) (Table 2). The mean BG was no different with either glucometer or iPro2 (Fig. 3). No significant differences were found in the standard deviation of BG values or the MAGE score. The number of patients experiencing at least one hypoglycaemic event was no different from one group to the other. However, iPro2 indicated a trend towards a difference in the time spent in hypoglycaemia during 1000 h of insulin infusion and the number of patients experiencing at least one hypoglycaemia event. The time spent in the target range was similar for the two groups. Twenty-three hypoglycaemic events were detected by glucometer during the study. In 16 of the 23 cases (69.6%), medication (e.g. 50 mL gravity fed infusion bag in 30 min, change in total parenteral nutrition bag, syringe changeover) had been administered during the three preceding hours through the line connected to the distal line of the central venous catheter.

**Table 2:** Summary of primary and secondary outcomes dealing with glycaemic variability

Parameters	Standard infusion line		Optimised infusion line		Between-group difference	
	*N*	Values	*N*	Values	Mean (95% CI)	*P* value
Glucometer						
GLI over 48 h, ITT analysis* ^†^ mean (95% CI)	86	5.37 (5.21; 5.53)	86	5.46 (5.27; 5.64)	−0.09 (−0.34; 0.16)	0.49^ǂ^
GLI over 48 h, complete case analysis* mean (95% CI)	79	5.35 (5.21; 5.49)	80	5.44 (5.26; 5.62)	−0.09 (−0.32; 0.13)	0.40^ǂ^
Capillary blood glucose average over 48 h, mg dl^−1^	79	134.8 ± 21.0 132.0 (117.0–149.0)	80	138.7 ± 26.2 133.5 (123.0–146.0)	–	0.58^#^
Capillary standard deviation over 48 h, mg dl^−1^	79	31.0 ± 13.3 29.0 (25.0–36.0)	80	32.5 ± 18.3 29.0 (22.5–37.5)	–	0.93^#^
MAGE over 48 h, mg dl^−1^	79	9.8 ± 6.2 9.0 (6.0–12.0)	80	10.1 ± 7.4 9.0 (5.0–12.0)	–	0.98^#^
At least one hypoglycaemia, *n* (%)	79	11 (13.9)	80	8 (10.0)	–	0.45^Ɨ^
At least one hyperglycaemia, *n* (%)	79	51 (64.6)	80	48 (60.0)	–	0.55^Ɨ^
CGM						
iPro2 glucose average over 48 h, mg dl^−1^	74	128.0 ± 21.3 124.5 (111.0–142.0)	70	133.5 ± 25.3 129.0 (117.4–139.0)	–	0.26^#^
iPro2 glucose standard deviation over 48 h, mg dl^−1^	74	23.3 ± 8.9 21.0 (18.0–25.0)	70	25.1 ± 12.3 21.0 (17.0–29.0)	–	0.75^#^
At least one hypoglycaemia detected by iPro2, *n* (%)	74	19 (25.7)	70	9 (12.9)	–	0.052^Ɨ^
Time spent in hypoglycaemia for 1000 h recorded with iPro2 (h)	74	9.7 ± 25.0 0.0 (0.0–1.74)	70	4.4 ± 14.8 0.0 (0.0–0.0)	–	0.059^#^
At least one hyperglycaemia detected by iPro2, *n* (%)	74	45 (60.8)	70	46 (65.7)	–	0.54^Ɨ^
Time spent in hyperglycaemia for 1000 h recorded with iPro2	74	64.3 ± 97.3 15.6 (0.0–97.2)	70	92.7 ± 181.1 13.9 (0.0–104.17)	–	0.78^#^
Time in target range recorded with iPro2	74	71 ± 26 80 (54–92)	70	72 ± 25 80 (64–78)	–	0.82^#^

Values are expressed as mean ± standard deviation and median (interquartile range) unless otherwise indicated

*ITT* Intended-to-treat

* Primary outcome: means and their 95% CI were calculated after log transformation of GLI values

^**†**^After the mean of 20 imputations to treat the missing values

^ǂ^Student *t* test

^#^Mann–Whitney *U* test

^Ɨ^Chi-square test

### Other outcomes

Table 3 reveals no differences in clinical outcomes between the groups. The length of stay in the HDU and in hospital was similar. Neither was there any difference in the insulin amount infused over 48 h between the standard infusion line [70.7 ± 25.9 IU (70.0)] and the optimised infusion line [78.0 ± 29.5 IU (78.0)] (*p* = 0.10), nor in the number of insulin flow rate changes over 48 h [6.3 ± 2.7 (6.0) vs 7.0 ± 3.2 (7.0), *p* = 0.13]. Insulin flow rates were between 1 and 5 ml h^−1^, with a mean flow rate at 2 ml h^−1^. However, there was a slight difference in the number of interventions on the infusion line, the number being high for the optimised infusion line (25.5 ± 8.3 vs. 22.9 ± 7.8, *p* = 0.039).

**Table 3:** Clinical outcomes and length of stay in perioperative HDU and in hospital

Parameters	Standard infusion line (*n* = 86)	Optimised infusion line (*n* = 86)	*P* value
Length of stay in HDU in days	6.2 ± 3.8 5.0 (4.0–7.0)	6.8 ± 5.6 5.0 (4.0–7.0)	0.65^#^
Length of stay in hospital in days	17.2 ± 12.5 13.0 (11.0–19.0)	21.0 ± 16.2 14.0 (11.0–25.0)	0.12^#^
Use of vasoactive drugs, *n* (%)	22 (28.2)	27 (34.2)	0.42^Ɨ^
Transfers to ICU, *n* (%)	2 (2.6)	5 (6.3)	0.44^Ɨ^
Deaths, *n* (%)	3 (3.8)	6 (7.3)	0.50^Ɨ^
Complications, number of patients with at least one complication, *n* (%)	50 (62.5)	57 (69.5)	0.35^Ɨ^

Values are expressed as mean ± SD and median (interquartile range) unless otherwise indicated

^#^Mann–Whitney *U* test

^Ɨ^Chi-square test

## Discussion

This is the first study, to our knowledge, to make a prospective comparison of two insulin infusion lines as well as of two glycaemic monitoring systems. The population consisted of high-risk patients undergoing, for the most part, oesophagectomy and extensive liver surgery. Such procedures leave a high risk of post-operative complications [22, 23], and so in our hospital, these patients are routinely treated in an HDU during the first 48 h of the post-operative period.

The first endpoint of the study concerned the lability index. There was no significant difference between the two infusion systems as regards the usual parameters to assess patients’ glycaemic variability. Glycaemic variability data can be compared to that already published. GLI values appeared to be similar to those reported by Dungan et al. in patients suffering type 2 diabetes and heart failure but inferior to Ali et al. data obtained in a population with sepsis [24]. MAGE values appeared slightly lower than those found by Hermanides et al. [25] in medical and surgical ICU patients. SD values were close to data published by Van den Berghe et al. [26] in 2006 concerning the same population.

The second endpoint of the study concerned hypoglycaemic events. A strong tendency to minimise hypoglycaemia was observed with optimised tubing. Data obtained from CGM showed an increase in the frequency of glycaemic events approaching the significance level just as in the time spent in hypoglycaemia during 1000 h of IIT. This trend was not linked with differences in patient outcomes. Reducing the incidence of potentially harmful events such as hypoglycaemia is clinically relevant. Baghshaw et al. [27] previously showed that early hypoglycaemia was associated with significantly higher ICU and hospital mortality rates, even after adjustment for available confounding factors. Moreover, blood glucose level around 7.0 mmol/l is associated with a measurable increase in the odds of survival, if hypoglycaemia is avoided [28]. In our study, CGM data analysis showed that the use of the optimised system with dedicated insulin infusion halved the number of patients experiencing at least one hypoglycaemic event. This result paves the way to a new study to assess this outcome with a greater number of patients.

This study is a follow-up to the retrospective study in a medical ICU published by Maury et al. in which authors compared two insulin infusion lines. The use of a dedicated line was linked to a significant decrease in the incidence of hypoglycaemic events. An important result of this study was confirmation of a trend towards a significant difference in the time spent in hypoglycaemia during 1000 h of IIT. The authors explained that their results were based on minimising insulin mass flow rate disturbances caused by multiple intravenous medications infused on the same infusion line as that of insulin. They showed that another drug had been administered through the insulin infusion line during the hour preceding the hypoglycaemic event in more than 8 of 10 events. In our study, more than two-thirds of hypoglycaemic events were correlated with the start of another drug administration on the infusion line in the 3 h preceding the event. This concords with our hypothesis at the beginning of this study based on in vitro works that showed the impact of common space volumes on mass flow rate disturbances when several medications are on the same infusion line [8, 11, 12].

The way to minimise the occurrence of hypoglycaemia lies in the infusion line. This is evident in the in vitro works cited above concerning the simultaneous infusion of several medications on the same infusion line [7, 11, 12]. The occurrence of hypoglycaemia is not linked with differences in morbidity and mortality between the groups.

An original aspect of this study was to use an innovative medical device, the Edelvaiss Multiline-8 which dedicates an infusion line to each drug. In this way, interactions between simultaneously infused medications are minimised. Its advantage has been demonstrated in preventing drug incompatibilities [13, 14] as well as mass flow rate disturbances both in vitro and on animals [7, 8, 14]. Our work supports its use in an HDU context and suggests a link between minimising interactions of simultaneous infused drugs and time spent in hypoglycaemia.

The difference between Maury’s study and ours cannot be accounted for by more intensive insulin treatment. It seems to be due to different recruitment, with ICU patients presenting gravity scores higher than our HDU population. High blood glucose seems to be correlated with a high APACHE II score and a more serious degree of disease such as a higher incidence of respiratory infection [29]. Glycaemic variability and hypoglycaemia therefore differ from ICU to HDU patients as the risk of severe hyperglycaemic stress response is higher in patients with organ failure than in those without [30]. Knowing that insulin bolus increases glycaemic variability and hypoglycaemia, the risk will be lesser in HDU patients [31]. Lower hyperglycaemic stress and less risk of bolus induced by other intravenous agents could explain the discrepancy with our results.

The other original aspect is the use of two methods to monitor patients’ glycaemic status. Control of the interstitial glucose level seems to increase sensitivity for detecting hypoglycaemic events as the real time spent in and out of the range of targeted glycaemic values can be calculated more precisely.

Better insulin dose adjustment is therefore possible, which would impact the time spent on therapeutic targets and patient survival [32]. Tight glycaemic control of critically ill patients has no effect on mortality but causes five times as much hypoglycaemia compared to mild or very mild control [33, 34]. Ways have to be found to limit hypoglycaemia occurrence, such as minimising infusion volume and monitoring glucose levels.

It can therefore be noted that the optimised infusion line is responsible for fewer hypoglycaemic events. The choice of the medical devices used to infuse insulin seems important for improving the safety of insulin infusion in a perioperative HDU. It could be worthwhile to develop a new clinical protocol integrating hypoglycaemic events as the primary endpoint and to compare patients’ outcome according to insulin infusion modalities.

## Abbreviations

ASA: physical status score defined by the American Society of Anesthesiologists
BG: blood glucose
BMI: body mass index
CGM: controlled glucose monitoring
CRFs: cardiovascular risk factors
GLI: glycaemic lability index
HDU: high dependency unit
ICU: intensive care unit
IIT: intensive insulin therapy
ITT: intended-to-treat
IU: international unit
MAGE: mean amplitude glycaemic excursions
SAPS II: simplified acute physiological score
SD: standard deviation
SOFA: sequential organ failure assessment
